# Binding of gephyrin to microtubules is regulated by its phosphorylation at Ser270

**DOI:** 10.1007/s00418-021-01973-2

**Published:** 2021-04-01

**Authors:** Lin Zhou, Eva Kiss, Rebecca Demmig, Joachim Kirsch, Ralph Alexander Nawrotzki, Jochen Kuhse

**Affiliations:** 1 Department of Histology and Embryology, Tongji Medical College, Huazhong University of Science and Technology, Wuhan, People’s Republic of China; 2 Department of Cellular and Molecular Biology, University of Medicine, Pharmacy, Science and Technology “G.E. Palade” of Târgu Mures, Târgu Mures, Romania; 3 University of Konstanz, Molecular Genetics, Konstanz, Germany; 4 Department of Anatomy and Cell Biology, Institut für Anatomie und Zellbiologie, University of Heidelberg, Lehrstuhl II, Im Neuenheimer Feld 307, 69120 Heidelberg, Germany

**Keywords:** Gephyrin, Phosphorylation, Microtubules

## Abstract

Gephyrin is a multifunctional scaffolding protein anchoring glycine- and subtypes of GABA type A- receptors at inhibitory postsynaptic membrane specializations by binding to the microtubule (MT) and/or the actin cytoskeleton. However, the conditions under which gephyrin can bind to MTs and its regulation are currently unknown. Here, we demonstrate that during the purification of MTs from rat brain by sedimentation of polymerized tubulin using high-speed centrifugation a fraction of gephyrin was bound to MTs, whereas gephyrin phosphorylated at the CDK5-dependent site Ser270 was detached from MTs and remained in the soluble protein fraction. Moreover, after collybistin fostered phosphorylation at Ser270 the binding of a recombinant gephyrin to MTs was strongly reduced in co-sedimentation assays. Correspondingly, upon substitution of wild-type gephyrin with recombinant gephyrin carrying alanine mutations at putative CDK5 phosphorylation sites the binding of gephyrin to MTs was increased. Furthermore, the analysis of cultured HEK293T and U2OS cells by immunofluorescence-microscopy disclosed a dispersed and punctuated endogenous gephyrin immunoreactivity co-localizing with MTs which was evidently not phosphorylated at Ser270. Thus, our study provides additional evidence for the binding of gephyrin to MTs in brain tissue and in in vitro cell systems. More importantly, our findings indicate that gephyrin-MT binding is restricted to a specific gephyrin fraction and depicts phosphorylation of gephyrin as a regulatory mechanism of this process by showing that soluble gephyrin detached from MTs can be detected specifically with the mAb7a antibody, which recognizes the Ser270 phosphorylated- version of gephyrin.

**Supplementary Information:**

The online version contains supplementary material available at 10.1007/s00418-021-01973-2.

## Introduction

Gephyrin is a highly evolutionarily conserved protein with multiple functions in neuronal and non-neuronal cells (Nawrotzki et al. 2012). It is well established as a major scaffolding protein for the organization of inhibitory glycine receptors (GlyRs) and type A GABA receptors (GABA_A_Rs) at postsynaptic sites (Tyagarajan and Fritschy 2014) in precise apposition to presynaptic terminals. The binding sequence motifs within the gephyrin E domain and large intracellular loop-domain of the GlyRs β subunit, as well as similar sequences in GABAARsα 1–3 subunits, have been characterized in detail (Maric et al. 2011). These interactions are thought to regulate the number and proportion of GlyRs and GABA_A_Rs at the postsynaptic membrane (Groeneweg et al. 2018) and to determine also the transport of gephyrin that is bound to the receptor-transport vesicles (Maas et al. 2006, 2009).

Several studies indicated the functional impact of microtubules (MTs) on gephyrin cluster formation. Gephyrin was initially identified as a GlyR-binding protein, and interestingly tubulin cofractionates with gephyrin upon GlyR purification by amino-strychnine affinity chromatography (Kirsch et al. 1991; Prior et al. 1992). Consistently, overlay and co-polymerization assays demonstrated high-affinity binding of gephyrin to purified polymerized tubulin (Kirsch et al. 1991). However, whether tubulin is indeed involved in the gephyrin-dependent receptor clustering remains controversial. MT disruption by various chemical agents strongly reduces the ability of both gephyrin and GlyRs to cluster at synaptic sites in early-stage-cultures of spinal cord neurons (Kirsch and Betz 1995), while Allison et al. found no effect of reduced MT assembly on gephyrin clusters in hippocampal neurons at DIV15 (Allison et al. 2000). Another study demonstrated that intracellular perfusion of the MT depolymerizing agent colchicine resulted in reduced immunofluorescence intensity of synaptic gephyrin/GlyR clusters and changes of GlyR functions, specifically a decrease of postsynaptic Cl^−^-currents using patch-clamp recordings in immature (DIV5-7, to a lesser extent, DIV10-12) rather than in mature (DIV15-17) stages of cultured hippocampal neurons (van Zundert et al. 2004). Hence, the impact of MTs may be relevant in gephyrin cluster formation of differentiating neurons, but less important for the maintenance of postsynaptic scaffold organization. Accordingly, during synaptogenesis, the retrograde movement of small co-localized GlyR and gephyrin immunoreactivities was found to occur at speed rates characteristic for MT-based fast transport mechanisms using live image recordings of cultured hippocampal neurons (Maas et al. 2006, 2009). Thus, taken together with data obtained from photoactivated localization microscopy and single-particle tracking (Specht et al. 2013), a picture emerges pointing to cytoplasmic gephyrin bound to receptor harboring vesicles that are transported along MTs near loci of inhibitory synapse formation. This suggests, that alterations of size, number or density of postsynaptic gephyrin clusters observed upon pharmacological treatments causing the disintegration of MTs might be the result of altered transport or disturbed local anchoring of gephyrin to MT or both. Therefore, a more detailed analysis of cellular mechanisms underlying the regulation of gephyrin- MT associations is needed.

Gephyrin binds directly or indirectly to an important number of other proteins in both neurons and non-neuronal cells (Groeneweg et al. 2018). One of these is collybistin (Cb), a guanine nucleotide exchange factor, that was identified by yeast two-hybrid screening (Kins et al. 2000). The interaction of Cb with gephyrin was found to result in the redistribution of gephyrin from cellular aggregates to submembrane micro-clusters in various non-neuronal cells. This function was dependent on the co-expression of the CbII-variant lacking a NH2-terminal Src-homology 3 (SH3) domain (Kins et al. 2000). The SH3 domain present in all other known Cb-splice variants was shown to provide auto-inhibition by interactions with the catalytic DH domain and a pleckstrin homology (PH) domain of Cb (Soykan et al. 2014). Apparently, to control gephyrin clustering at the postsynaptic compartment as well as GABA_A_R recruitment to synapses, the SH3 domain-dependent interaction of Cb with additional membrane proteins, such as neuroligin-2 and -4 (NL2, NL4) (Poulopoulos et al. 2009; Hoon et al. 2011) or the α2-subunit of GABA_A_Rs (Tretter et al. 2008; Saiepour et al. 2010) is required, specifically for inducing an open and active conformation of Cb. Consistently, the analysis of Cb knock out mice revealed impaired GABAergic transmissions and altered synaptic plasticity in the mouse hippocampus and a restricted number of other brain areas (Papadopoulos et al. 2007). The functional importance of Cb in humans became evident by the identification of a number of mutations in the human Cb gene localized on the X chromosome causing X-linked mental retardation and epilepsy (Alber et al. 2017).

In line with these findings, we reported the complete loss of synaptic gephyrin clusters upon Cb knock down experiments in cultured rat hippocampal neurons (Kuhse et al. 2012). In addition, these experiments evidenced that the loss of Cb in these cells also resulted in a complete loss of gephyrin phosphorylation at Ser270, a site that is specifically recognized by the phospho-specific anti-gephyrin antibody mAb7a and correlated with reduced GABA_A_R clustering (Kuhse et al. 2012). The functional implications of this phosphorylation for the Cb-dependent postsynaptic synapse organization, however, remain to be established.

It is overall accepted that different forms of posttranslational modifications of gephyrin including sumoylation, acetylation, nitrosylation, palmitoylation, and phosphorylation might modulate gephyrin activity (Ghosh et al. 2016; Groeneweg et al. 2018). Specifically, gephyrin phosphorylation at different sites induced by different kinases such as glycogen synthase kinase 3 β(GSK3β) (Tyagarajan et al. 2011), cyclin dependent kinase 5 (CDK5) (Kuhse et al. 2012; Zacchi et al. 2014; Kalbouneh et al. 2014; Kiss et al. 2020) and Ca^2+^/calmodulin-dependent protein kinase II (CaMKII) (Flores et al. 2015) is an important factor determining the number, size and protein density of postsynaptic gephyrin scaffolds as well as the binding of different GlyRs and GABA_A_Rs. However, whether the phosphorylation of gephyrin or other modifications contribute to the gephyrin-tubulin interaction is yet not known.

Here, we addressed primarily this question and tested whether gephyrin could be copurified with MTs from rat brain tissue and the gephyrin-MT association correlates with the Cb-dependent phosphorylation of gephyrin at Ser270. Our results show that phosphorylation of gephyrin at Ser270 indeed contributes to the detachment of gephyrin from MTs in both neuronal and non-neuronal cells.

## Materials and methods

### Purification of tubulin from rat brain

The purification protocol was performed with some modifications as described (Sloboda 2015). Briefly, fresh brains from adult rats (1.5–2 gr) were minced and gently homogenized by a Potter homogenizer in 4 ml cold P^+^-buffer (PIPES/KOH 100 mM, pH 6.85, EDTA 0.1 mM, EGTA 2 mM, MgCl_2_ 0.5 mM, proteinase inhibitors mixture (complete, Roche Applied Science), 1 mM ATP, 0.1% mercaptoethanol). The brain homogenate was clarified by centrifugation at 12,000× g, 4 °C, for 15 min. The supernatant was taken and subjected to a further centrifugation step at 33,000× g, 4 °C, for 60 min ("first cold spin"). The pellet was discarded and the supernatant was adjusted to final concentrations of 1 mM GTP, 1.5 mM ATP and 4 mM MgCl_2_. The mixture was incubated at 37 °C for 1 h after adding half volume of pre-warmed (37 °C) glycerol. For the sedimentation of polymerized tubulin, the solution was centrifuged at 53,000× g for 2 h ("first warm spin"). After centrifugation, the supernatant was stored at −80 °C and the pellet was resuspended in the same volume of cold P^+^-buffer as the supernatant. After being left on ice for 30 min, the resuspended pellet fraction was aliquoted into 4 volumes and subjected to the second cold spin at 53,000× g, 4 °C for 1 h. The pellet was stored at −80℃ and the supernatant was mixed with GTP, ATP and MgCl_2_ followed by glycerol at the aforementioned concentration. After incubation at 37 °C for 1 h, the mixed solution was subjected to the second warm spin at 53,000× g, 37 °C for 1 h. Both the supernatant and pellet were collected. The pellet fraction with enriched MTs was resuspended and later used for the MT co-sedimentation assay. The protein concentration of the solutions was determined by the BCA protein assay (kit from Pierce).

### Harvest of HEK293T cell lysates

HEK293T cells were used to express recombinant gephyrin (P1) (Prior et al. 1992). Cells were cultured in 10 cm dishes and harvested 2 days after transfection with 15 μg plasmid DNA. The cells were washed once with cold DPBS. 1 ml lysis-buffer (50 mM Tris–HCl, pH7.5, 150 mM NaCl, 0.1% Nonidet P-40, 0.25% SDS, 2 mM sodium orthovanadate, and protease inhibitor mixture) was added and the cells were scraped from the dish on ice and transferred into 1.5 ml tubes. Then the cells were homogenized mechanically in a Potter homogenizer on ice and centrifuged for 30 min at 13,500× g at 4 °C. The supernatant was transferred in new tubes and kept on ice until using or stored at −80℃ for later use.

### MT co-sedimentation assay

Purified tubulin samples and/or HEK293T extracts with recombinant gephyrin were mixed with MES buffer and DTT (final concentration: 0.1 M MES, pH 6.6, 1 mM EGTA, 1 mM MgSO_4_ and 1 mM DTT). For efficient tubulin polymerization 2 mM GTP and 20 μM paclitaxel were added. For some experiments, extracts with recombinant gephyrin or gephyrin/mutant-gephyrin and CbII cellular tubulin was polymerized under the same conditions. The mixture was incubated at 37 °C for 20 min followed by centrifugation for 20 min at RT at 12,000× g and the supernatant was subjected to a further centrifugation step for 40 min at 22 °C at 100,000× g. Both pellet and supernatant were collected for SDS gel electrophoresis. For purified tubulin preparation, one aliquot of the pellet after second warm spin was unfrozen and resuspended in 200 μl MES buffer with DTT. BCA assay was applied to detect the concentration of tubulin in the suspension. The purified tubulin was left 30 min on ice, then subjected for MT co-sedimentation assay. HEK293T cell lysate was thawed on ice until it was completely unfrozen and used for MT co-sedimentation assay. In general, a total volume of 50 μl (in some cases 30 μl) assays with varying concentrations of purified tubulin and gephyrin-extracts was subjected to co-sedimentation assays.

### SDS-gel electrophoresis and immunoblot analysis

The equivalent volumes of supernatant and resuspended pellet solution of each centrifugation step of the tubulin purification or after MT co-sedimentation assay were mixed with 4 × loading buffer (250 mM Tris pH 6.8, 8% SDS, 40% glycerin, 0.04% bromphenol blue, and 20% β-mercaptoethanol) and denatured by heating for 5 min at 95 °C. The denatured samples were separated by 10% SDS-PAGE, then stained with Coomassie dye or directly transferred to a nitrocellulose membrane. Membranes were probed with polyclonal goat anti-gephyrin antibody R-20 (1:1000, Santa Cruz Biotechnology), mouse monoclonal anti-phospho-specific gephyrin antibody mAb7a (1:500, Synaptic Systems, Göttingen, Germany), mouse monoclonal anti-α-tubulin antibody DM1α (1:100,000, Sigma), polyclonal chicken anti-collybistin antibody (1:50) and mouse anti-tau antibody (1:10,000, Invitrogen). An appropriate secondary antibody conjugated to HRP (Millipore) was used at a dilution of 1:10,000 for detection. The secondary antibodies were detected using ECL-Prime detection kit (Amersham Biosciences). After suitable exposure times, hyperfilms (Amersham Bioscience) were developed and signals were scanned and analyzed using Fiji software. The membranes were stripped in stripping buffer (100 μM β-mercaptoethanol and 2% SDS, 62.5 mM Tris–HCl, pH 6.7) at 50 °C for 30 min after the incubation of an antibody. The membranes were rinsed three times with TBST, blocked again, and used for further antibody detection.

### Construction of expression plasmid

DNA fragments encoding the complete coding sequence of rat gephyrin (P1 clone) (Prior et al. 1992) were amplified by PCR. N-terminally 6-histidine-tagged gephyrin-coding PCR products with flanking NotI and XhoI sites were cloned into vector pcDNA5/FRT/TO (Invitrogen). Site-directed mutagenesis of His6-gephyrin at Ser198, Ser200, Ser270 was performed using the QuickChange Lightning mutagenesis kit from Stratagene following the supplier’s instructions. Both expression plasmid and mutants were verified by sequence analysis. CbI and CbII expression plasmids were described elsewhere (Kins et al. 2000).

### Cell culture and transfection

HEK293T cells were cultured in 10% DMEM medium and passaged every 4 days. Human osteosarcoma cell line U2OS cells were grown in 10% McCoy’s 5a medium and passaged every 3 days. Cells were transfected with gephyrin expression plasmid or co-transfected with gephyrin-/mutant gephyrin- and CbII-expression plasmids. The transfection was performed with 15 μg DNA, 57.5 μl polyethyleneimine (PEI, Sigma) and up to 750 μl Opti-MEM® (Invitrogen) for a 10 cm dish, 2 μg DNA, 2 μl PEI and up to 50 μl Opti-MEM for a 24-well plate. The transfection mixture was kept at RT for 30 min before added to the adherent cells for 4 h incubation in a humidified incubator at 37 °C and 5% CO_2_, followed by a change of prewarmed culture medium. The incubation was repeated under the same conditions until the cells were harvested or fixed for staining. As transfection control, additional cells were transfected with a vector expressing gephyrin combined with a C-terminal GFP.

### Immunocytochemistry

HEK293T and U2OS cells were seeded on glass coverslips in 24-well plates at a density of 1 × 10^5^/well and 5 × 10^4^/well, respectively. Transfected and non-transfected cells were washed with 1 × ice-cold PBS (with Ca^2+^ and Mg^2+^), then fixed in 4% (w/v) paraformaldehyde in PBS at RT for 10 min. Blocking and permeabilization were performed in 5% (v/v) normal horse serum, 1% (v/v) bovine serum albumin and 0.1% (v/v) Triton X-100 for 1 h. Fixed cells were incubated with primary antibodies, mouse anti-phospho-gephyrin mAb7a (1:200), polyclonal rabbit anti-gephyrin Ab-175 (1:100), mouse anti-tubulin DM1α (1:10,000), chicken anti-Cb (1:1000) antibody in the same solution used for blocking, except Triton X-100 was omitted. Appropriate secondary antibodies were coupled to Alexa488 (1:100), Cy3 (1;1000) or cy5 (1:500). Phalloidin-TRITC (1:3000, Molecular Probes) was used in some triple labeling experiments. Where necessary, DAPI (1:50) was used to counterstain the nuclei. Images were collected by a confocal Leica TCS SP8 microscope (Leica Microsystems CMS GmbH, Mannheim, Germany) using a HC PL APO CS2 63.0 × 1.40 oil objective or with a STEDYCON microscope from Abberior Instruments GmbH.

### Electron microscopy

A) Nearly confluent HEK 293 cells were fixed with 2.5% glutaraldehyde containing 2.5% polyvinylpyrrolidone (PVP, MW 25.000, Polydon, Merck) and 0.05% CaCl_2_ in 0.1 M sodium cacodylate buffer (pH 7.6) for 30 min. For enhancement of membrane staining the cells were incubated for 60 min in alkaline 3,3ˊ-diaminobenzidine (DAB) (pH10.0). The samples were then postfixed with buffered 1.5% osmium tetroxide (OsO_4_) in cacodylate buffer containing 1.5% potassium ferrocyanide for 20 min followed by two additional osmifications, each for 20 min: (1) buffered 1.5% OsO_4_ in cacodylate buffer and (2) buffered 1.5% OsO_4_ in cacodylate buffer containing 3% potassium chromate. The cells were rinsed in both cacodylate and 0.05 M maleate buffer, stained with buffered 1% uranylacetate in maleate buffer, dehydrated in a series of graded ethanols, transferred to 2-hydroxypropylacrylat (HPMA), and finally processed for Epon embedding. Ultrathin sections were prepared and stained with lead citrate and analyzed using a Zeiss EM10 electron microscope.

B) The HEK293 cells were fixed with 4% paraformaldehyde containing 2% PVP in 0.1 M phosphate buffer (PBS, pH 7.2) for 30 min, rinsed in PBS followed by 0.1 M Pipes-buffer and incubated in 0.75% tannin in Pipes buffer for 25 min. The samples were then rinsed in Pipes- and maleate buffer and stained with 1% uranylacetate for 20 min. After removal from the petri-dish bottom with a sharp razor blade the cells were dehydrated in graded ethanols (−20 °C), transferred to ethanol-LR-white (LRW) mixtures and embedded in LRW. Ultrathin sections were incubated for 24 h with a peptide antibody (rabbit) against the amino acids 521–538 of rat gephyrin (X66366) visualized by 15 nm protein A-gold on sections that were stained with uranylacetate and lead citrate and analyzed with the EM10.

### Blue-native polyacrylamide gel electrophoresis (BN-PAGE)

BN-PAGE analysis was performed as described (Schägger and von Jagow 1991). Gels were polymerized using ammonium persulfate (APS) and TEMED. After polymerization, the gels were stored overnight at 4 °C. Per gel lane, 100 μg of cytosol were applied after dilution with BNP-sample buffer (750 mM aminocaproic acid, 50 mM Bis–tris/HCl, 0.5 mM EDTA and 5% (v/v) glycerol, pH 7.0) in a ratio of 1:4. Cathode and anode buffers used for the separation consisted of 50 mM Tricine, 15 mM Bis–tris, 0.02% (w/v) Coomassie G250, pH 7.0, and 50 mM Bis–tris adjusted to pH 7.0 with HCl, respectively. The lanes were loaded horizontally onto 12.5% SDS-PA gels and electrophoresed. NativeMARK™ protein standard (Invitrogen) was used for the evaluation of protein size.

### Data analysis

Where appropriate, the data were presented as means ± standard deviations (SD). The statistical significance was measured by Student's t-test. Calculations were performed with Prism Software. P values of less than 0.05 were considered statistically significant.

## Results

### Gephyrin is associated with MTs purified from rat brain.

**Fig. 1:**
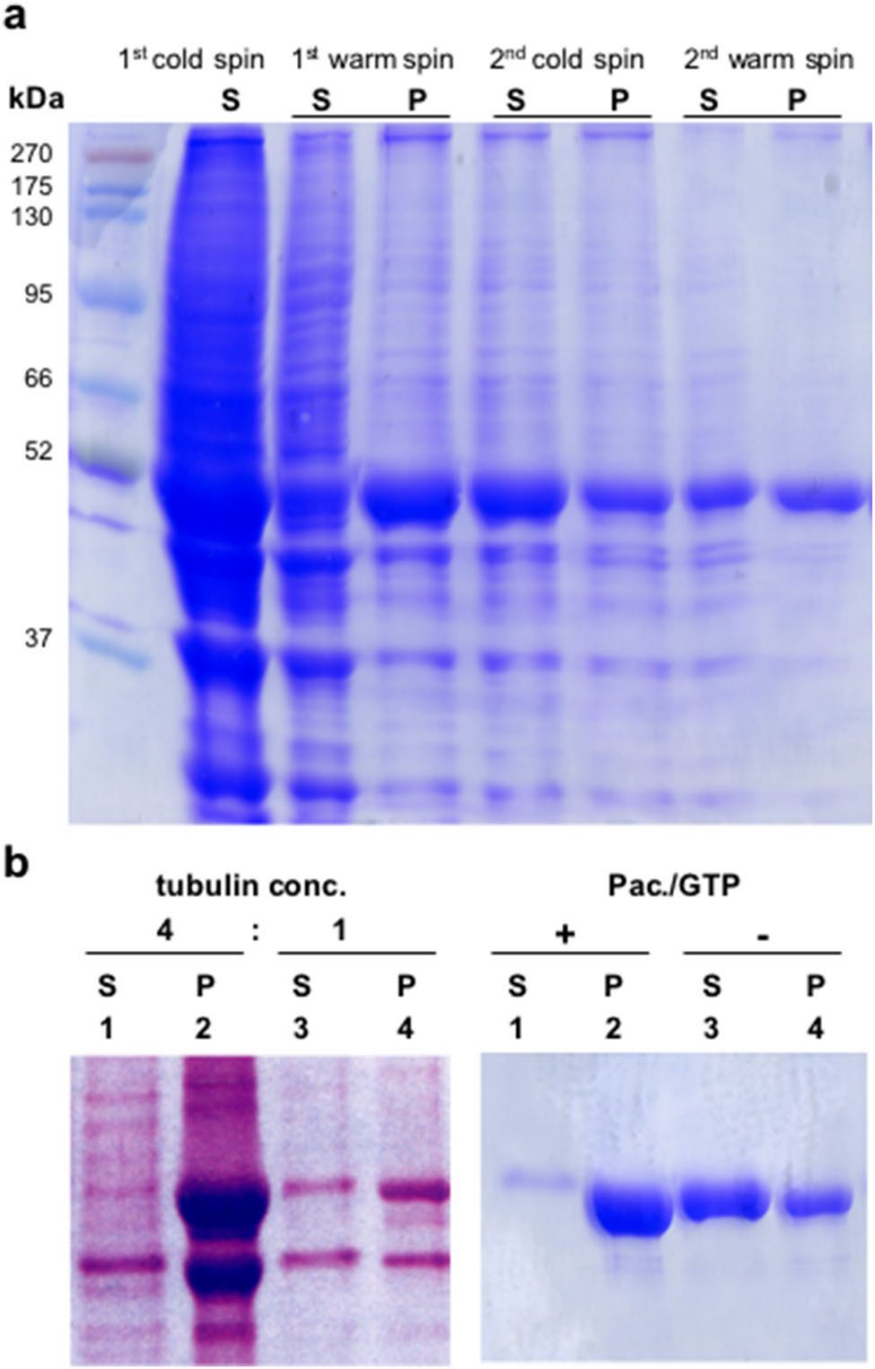
Purification of MTs from rat brain protein extracts. **a** Tubulin purified from rat brain was analyzed on SDS-PAGE gels stained with Coomassie blue. MTs were separated by high speed-sedimentation after two cycles of polymerizations from soluble proteins (“warm spin”). Tubulin is present in both, the soluble (S) and the pellet fractions (P). **b** Tubulin polymerization is augmented by a fourfold increase of the tubulin concentration in the sedimentation assay. MT assembly is also promoted by the presence of GTP and paclitaxel. Purified MTs after the 2nd warm spin were used for the experiment. Note, the ratio of tubulin content in the pellet compared to supernatant is increased in the presence of paclitaxel and GTP. M, protein marker; P, pellet; S, supernatant. Representative images of three independent experiments are shown

In a previous study, we demonstrated the co-sedimentation of MTs with affinity-purified GlyRs attached to gephyrin (Kirsch et al. 1991). Furthermore, in an additional assay we overlayed membrane bound gephyrin with depolymerized MT. After induction of tubulin polymerization MT binding to gephyrin could be detected (Kirsch et al. 1991). To study in more detail the interaction between gephyrin and MTs without the interference of GlyR subunits, we purified MTs from rat brain using the temperature cycling method (Sloboda 2015). We employed two cycles of MT depolymerization (4 °C) and polymerization (37 °C) followed each time by sedimentation of polymerized MTs by centrifugation (cold- and warm-spin, respectively). As shown in Fig. 1a, the ~50 kD β-tubulin protein band was predominantly present in the supernatant after each "cold spin" representing depolymerized soluble tubulin and was sedimented into the pellet after conditions allowing polymerization of tubulin at 37 °C (“warm spin”). After a second warm spin cycle tubulin was highly enriched in the pellet fraction and only minor amounts of other proteins could be seen in Coomassie staining of polyacrylamide gels (Fig. 1a).

Next, we proved the conditions for co-sedimentation assays with this enriched tubulin pellet fraction. The self-assembly of MTs is known to depend on several experimental parameters. Thus, we performed a sedimentation assay with different tubulin concentrations. As expected, high concentrations of tubulin in the sedimentation assay resulted in a more efficient MT polymerization (Fig. 1b). GTP and the plant-derived agent paclitaxel are known to promote MT-assembly. Thus secondly, we performed the co-sedimentation assay in the presence or absence of these substances. As shown in Fig. 1b the formation of MTs was strongly increased as indicated by the much higher tubulin amount found in the pellet fraction compared to the soluble fraction in the presence of paclitaxel and GTP.

To prove the binding of gephyrin to MTs we next employed the purified polymerized tubulin and protein extracts with gephyrin expressed from HEK293T cells for co-sedimentation assays routinely used to study the binding of MT associated proteins (MAPs) (Alberico et al. 2017).

**Fig. 2:**
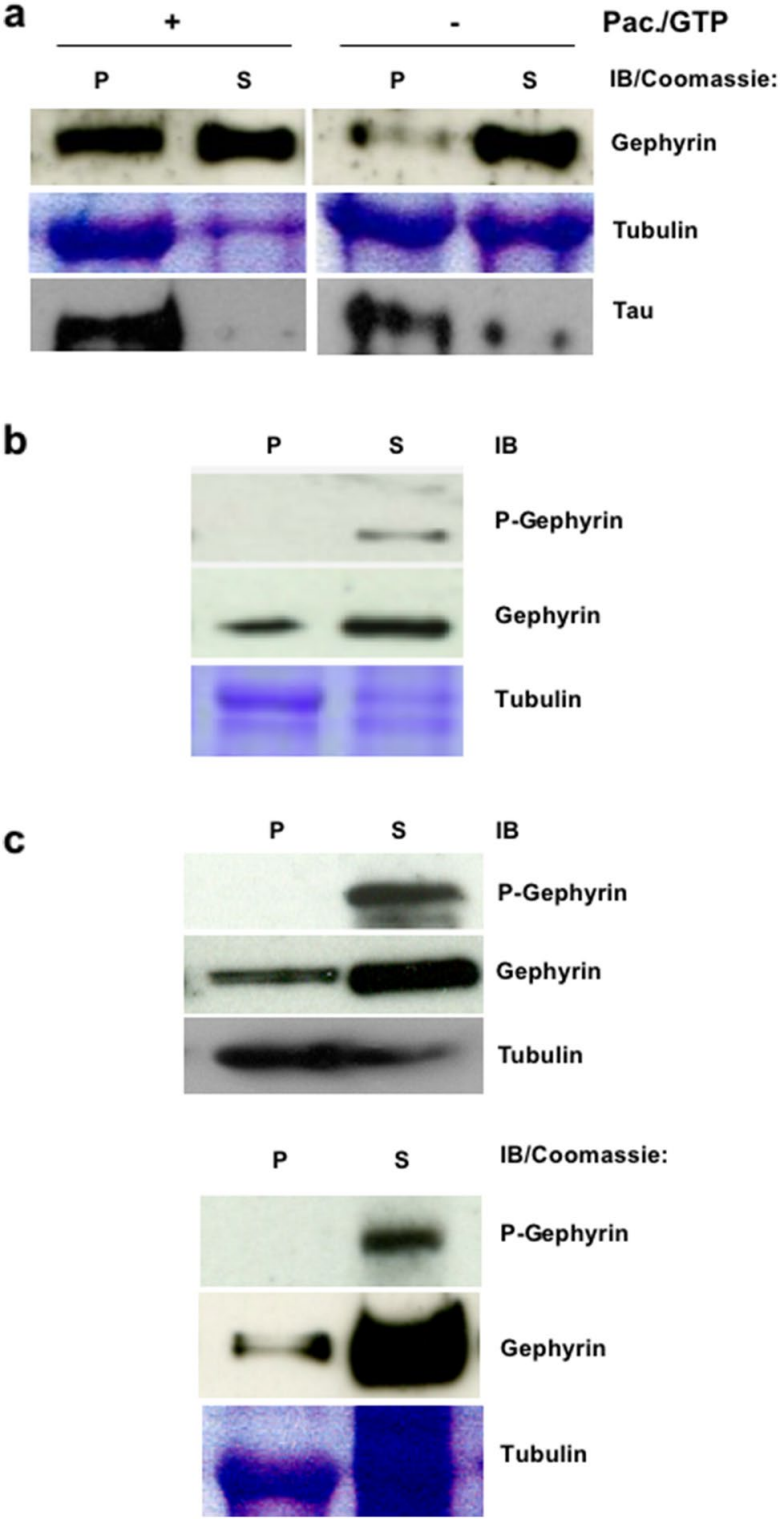
Gephyrin phosphorylated at Ser270 does not bind to the polymerized MTs. **a** Sedimentation of gephyrin with MTs in the co-sedimentation assay. Tubulin enriched after two cycles of polymerization was used for this assay. Sedimentation of gephyrin detected by R-20 antibody was largely improved upon increased MT polymerization in the presence of GTP and paclitaxel (Pac). Tau sedimentation was used as a positive control. **b** Detection of gephyrin with the phospho-specific anti-gephyrin antibody mAb7a (P-gephyrin) in the gephyrin—MTs co-sedimentation assay. Gephyrin phosphorylated at Ser270 does not bind to polymerized MTs during tubulin purification. A representative image of three independent experiments is shown. **c** Tubulin purification from different rat brains consistently showed the detection of the mAb7a positive gephyrin (P-gephyrin) exclusively in the supernatant fraction of soluble tubulin (S), while gephyrin detected by R-20 was distributed in both the supernatant fraction of soluble- and the pellet fraction of polymerized- tubulin. P, pellet; S, supernatant; IB, immunoblot. Two representative images of three independent experiments are shown

Interestingly, under experimental conditions that favor MT polymerization (GTP and paclitaxel) the amounts of gephyrin found to be bound to MTs were also higher than under conditions with lower tubulin polymerization rate (Fig. 2a). Altogether, these findings supported the binding of gephyrin to polymerized MTs rather than to soluble mono- or dimeric tubulin. As a positive control, we probed the same membrane with an antibody directed against the MT-associated protein tau. As seen in Fig. 2a in the presence of GTP/Pac tau was almost completely sedimented with the polymerized tubulin.

### Gephyrin detected by the phospho-specific antibody mAb7a does not bind to polymerized MTs in vitro

The binding of the major MAPs to MTs is mainly regulated by phosphorylation (Ramkumar et al. 2018). Therefore, we addressed the question whether the not MT-bound state of gephyrin might rely on differences in phosphorylation. For this aim, we took advantage of a phospho-specific anti-gephyrin antibody (mAb7a) detecting the phosphorylation of gephyrin at Ser270 (Kuhse et al. 2012). Indeed, performing a co-sedimentation assay (in the presence of GTP and paclitaxel) phosphorylated gephyrin detected by mAb7a was exclusively present in the supernatant (Fig. 2b).

To further confirm this finding we analyzed also the distribution of endogenous gephyrin in the soluble and non-soluble protein fractions of the first "warm spin" step during tubulin purification by WB. As shown in Fig. 2c this analysis supported the data from co-sedimentation experiments, revealing the exclusive detection of gephyrin phosphorylated at Ser270 by mAb7a in the soluble supernatant fraction (*n* = 3). The selective binding of non-phosphorylated gephyrin to MTs suggests that phosphorylation of gephyrin at Ser270 may be an important mechanism for detaching gephyrin from MTs in neuronal tissue similar to the detachment of tau from MTs upon hyperphosphorylation.

### Gephyrin binding to MTs is impaired by CbII_(-SH3)_- fostered phosphorylation

**Fig. 3:**
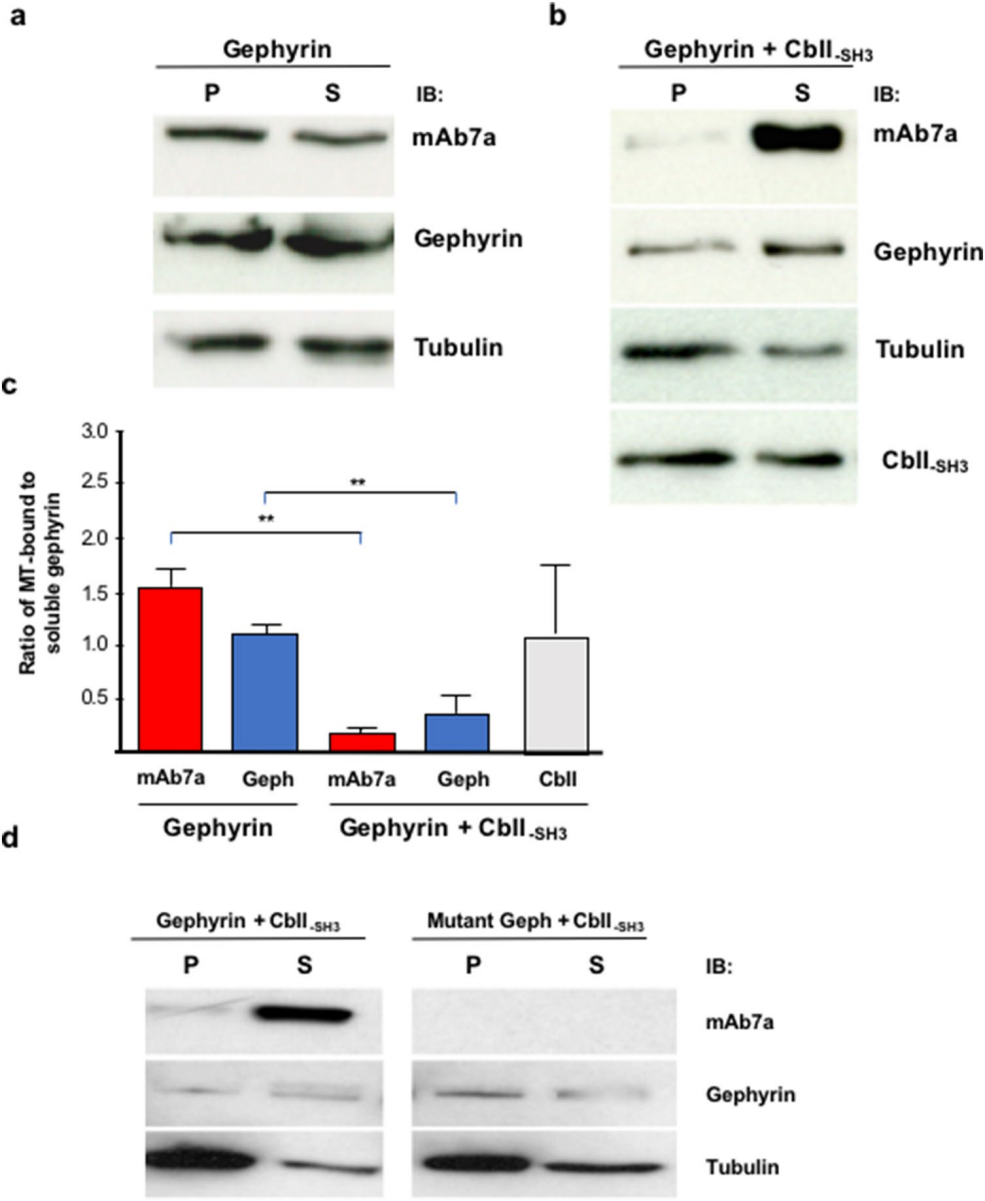
Gephyrin binding on MTs is modified by CbII(-SH3). Representative Western blots of gephyrin—MT co-sedimentation assays performed with lysates of HEK293T cells (**a**) transfected with gephyrin. Gephyrin expressed in HEK293T cells is already phosphorylated at a low basal level (Kuhse et al. 2012) and therefore detected by the mAb7a antibody in both, the pellet and soluble fraction, similar to the detection pattern with the non phospho-specific anti-gephyrin antibody R-20. Note the low efficiency of MT assembly in the HEK293T cell lysates due to the low level of endogenous tubulin concentrations. **b** When co-expressing gephyrin and CbII(-SH3) the level of phosphorylated gephyrin detected with mAb7a was strongly decreased in the pellet fraction (P). Gephyrin detected by R-20 showed also a higher signal intensity in the soluble protein fraction. The signal intensity of Cb immunoreactivity was equal for pellet and supernatant. **c** Quantification of experiments (*n* = 5) shown in B. The ratio of averaged band-intensities measured for the pellet or supernatant fractions was standardized to the tubulin (DM1α) signal. Note the decreased pellet-to-supernatant ratio of the R-20 signal in the lysates from cells co-transfected with gephyrin and CbII(-SH3). P, pellet; S, supernatant. **d** Alanine-mutations of putative phosphorylation sites within gephyrin abolished Cb mediated regulation of gephyrin binding to MTs as evidenced by immunoblot analysis after gephyrin and MT co-sedimentation assay using lysates of HEK293T cells co-expressing CbII(-SH3) and gephyrin or CbII(-SH3) and triple mutant gephyrin at Ser-198, Ser-200 and Ser-270 sites. Gephyrin mutated at Ser270 is not detected by the phospho-specific antibody mAb7a. Note that the distribution of gephyrin detected with antibody R-20 was shifted to the pellet fraction, with co-expression of CbII(-SH3) and mutant gephyrin. P, pellet; S, supernatant; IB, immunoblot

Cb is a gephyrin binding protein (Kins et al. 2000) essential for gephyrin clustering and GABA_A_R recruitment to synapses in defined areas of the brain (Papadopoulos et al. 2007). Until now, four splice variants of Cb have been reported (Saiyed et al. 2007). Three of these (CbI-III) differ in their C-terminal sequences and harbor a SH3-domain near the N-terminus. Interestingly, one isoform (CbII) exists with and without this SH3-domain (Kins et al. 2000; Saiyed et al. 2007; Körber et al. 2012), differentially affecting gephyrin clustering in hippocampal neurons (Poulopoulos et al. 2009; Hoon et al. 2011). Previously we have shown that CbII is involved in the regulation of gephyrin phosphorylation in both hippocampal neurons and HEK293T cells upon heterologous expression (Kuhse et al. 2012). However, the functional impact of this Cb mediated gephyrin phosphorylation is not completely clear. To analyze whether the binding of gephyrin to MTs might be affected by the Cb-dependent phosphorylation of gephyrin, MT co-sedimentation assays were performed with lysates of HEK293T cells expressing recombinant gephyrin alone, or co-expressing recombinant gephyrin and CbII_(-SH3)_. Previously, we have demonstrated that CbII_(-SH3)_ induces a strong CDK5-dependent gephyrin phosphorylation which can be detected by the mAb7a antibody (Kuhse et al. 2012). The soluble and non-soluble protein fractions were analyzed by immunoblotting using mAb7a and R-20 anti-gephyrin antibodies. In lysates of HEK293T cells expressing recombinant gephyrin alone, the distribution of gephyrin between soluble and insoluble fractions as detected with the R-20 antibody was similar to the findings in brain extracts. The polymerization of only endogenous tubulin from the HEK293T-cell extracts, might explain the somewhat lower efficiency of MTs sedimentation in these experiments (Fig. 3a). Prolonged exposure of the Western blots with the mAb7a antibody revealed signals also in the pellet fraction, due to a low binding affinity of mAb7a to recombinant gephyrin expressed in HEK293T cells with no or only low level of phosphorylation as shown previously (Kuhse et al. 2012). By contrast, when using extracts from HEK293T cells co-expressing gephyrin and CbII_(-SH3)_ we observed a robust increase of the mAb7a signal intensity in the supernatant fraction (Fig. 3b), whereas only a very weak mAb7a signal was detected in the insoluble MTs bound fraction. The gephyrin immuno-signal detected by the R-20 antibody on the same membrane was also stronger in the soluble protein fraction than in the MT-containing pellet, indicating an overall reduced binding of gephyrin to MTs in the double transfected cells.

The ratio of either mAb7a or R-20 immunoreactivities in the pellet compared to the supernatant fractions was determined by quantifying the intensities of the Western blot signals of the single experiments (*n* = 5). As shown in Fig. 3b, c the ratio of the mAb7a signal in the insoluble to soluble fraction was significantly lower in the presence of CbII_(-SH3)_ (0.170 ± 0.062) than in the absence of CbII_(-SH3)_ (1.50 ± 0.224). The increased amount of gephyrin in the supernatant was also detected with the R-20 antibody albeit not as distinctive as with the mAb7a antibody (Fig. 3b, c).

To confirm and gain further details about the involvement of gephyrin phosphorylation in the regulation of gephyrin-MT interaction, we employed MT co-sedimentation assay with extracts from HEK293T co-expressing CbII_(-SH3)_ and gephyrin with triple alanine-mutation at Ser198, Ser200, and Ser270, which are putative CDK5 phosphorylation sites and thought to be involved in CbII_(-SH3)_ -dependent phosphorylation of gephyrin. As shown in Fig. 3d, in agreement with the previous results, the co-expression of gephyrin and CbII_(-SH3)_ resulted in a predominant occurrence of phosphorylated gephyrin in the supernatant. However, analyzing the distribution of the mutant gephyrin^S198-S200-270A^ the main gephyrin immunoreactivity detected by R-20 was shifted to the pellet fraction, suggesting an increased binding of the probably non-phosphorylated gephyrin to MT. As expected, the detection of gephyrin with mAb7a was negative in the mutated gephyrin expressing cell fractions, this being in concordance with our previous findings that the mutation at Ser270, abolishing phosphorylation at this site, reduces strongly the binding affinity of the mAb7a antibody (Kuhse et al. 2012) (Fig. 3d).

These results supported our hypothesis that gephyrin phosphorylated at Ser270 is preferentially soluble and not bound to polymerized MTs and suggested that the phosphorylation of gephyrin by CDK5 or GSK3β at Ser270 (Tyagarajan et al. 2011; Kuhse et al. 2012) might represent an important mechanism which regulates the transient binding of gephyrin to MT.

### The oligomerization of endogenous gephyrin in HEK293T and U2OS cells is similar to gephyrin oligomerization in brain and liver tissue

Gephyrin is known to form oligomers due to the trimerization of its N-terminal G-domains and dimerization of its C-terminal E-domain (Saiyed et al. 2007; Kasaragod and Schindelin 2019). Thus, the association with MTs might be due to the binding of gephyrin oligomers rather than monomeric gephyrin to the polymerized tubulin. To analyze which oligomeric states gephyrin adapts in different cellular contexts we applied protein extracts from HEK293T-, U2OS cells and liver to blue-native PAGE and determined the apparent molecular mass of gephyrin detected by Western blotting. As shown in Suppl. Figure 1, one major gephyrin band migrating above a reference protein of 720 kD was detected in protein extracts from HEK293T and U2OS cells, comparable to a reference band from liver tissue that was shown previously to migrate identical to gephyrin identified in brain extracts (Nawrotzki et al. 2012). This size (> 720 kD) is generally believed to represent hexameric gephyrin whereas the faster migrating band (> 480 kD) is considered to correspond to gephyrin trimers (Herweg and Schwarz 2012).

This observation suggests that gephyrin expressed in different tissues and cell lines such as HEK293T and U2OS, shares similar biochemical properties determining the oligomerization behavior of this multifunctional protein. Moreover it is tempting to speculate that in non-neuronal tissue as well as in brain tissue these gephyrin oligomers bind to MTs rather than monomeric gephyrin, a hypothesis that is supported by the finding that the phosphorylation-dependent detachment of gephyrin from MT is very similar in neuronal and non-neuronal cells.

### Dispersed gephyrin oligomers in HEK293T cells are not detected with mAb7a

**Fig. 4:**
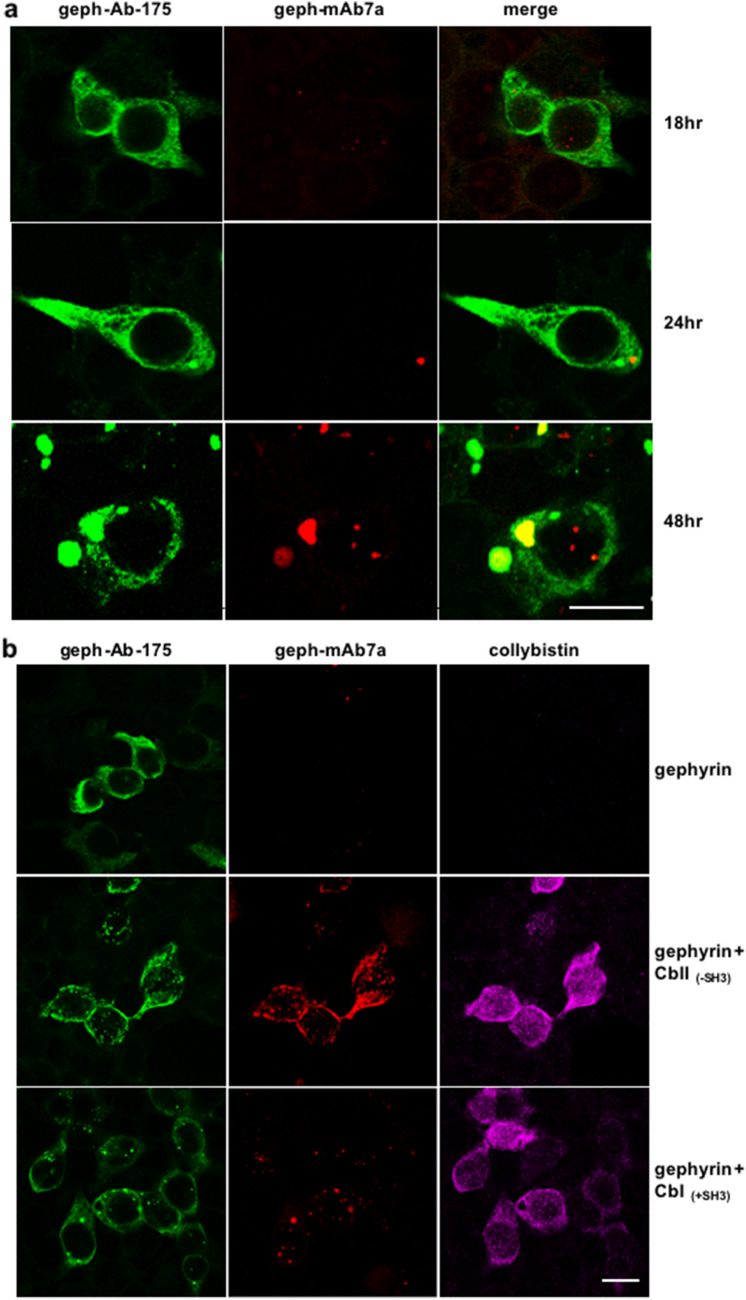
Cytoplasmic dispersed gephyrin is not detected by the phospho- specific (Ser270) mAb7a antibody in HEK293T cells. **a** Subcellular distribution of gephyrin in HEK293T cells. Gephyrin-transfected cells were fixed 18 h, 24 h and 48 h after transfection followed by immunofluorescent staining using the anti-gephyrin Ab-175 and phospho-specific anti-gephyrin mAb7a antibody. Dispersed Ab-175 immunoreactivity was detected throughout the cytoplasm at all examined timepoints. Ab-175 positive intracellular aggregates were only barely detected 18 h and 24 h after transfection, whereas, numerous and larger blobs were observed after 48 h. Importantly, almost no mAb7a immunoreactivity co-localized with the Ab-175 detected dispersed gephyrin, although, in cells fixed 48 h after transfection a certain fraction of blobs stained also with mAb7a. In addition, small mAb7a positive structures co-localizing with nuclear DAPI were also observed (DAPI staining not shown). Scale bar is 15 μm. **b** CbII_(-SH3)_ expression re-localized gephyrin to the cell membrane. HEK293T cells expressing gephyrin were co-transfected with different Cb isoforms and analyzed after 24 h. Upon co-expression of gephyrin with CbII lacking the SH3 domain, gephyrin detected with Ab-175 was routed from the cytoplasm to the plasma membrane. Strikingly, phosphorylated gephyrin detected with mAb7a showed strong immunoreactivity overlapping with Ab-175 staining. Cb immunoreactivity identified cells co-expressing gephyrin with different Cb isoforms. Upon co-transfection of gephyrin with CbI (with SH3 domain), smaller and larger blobs were detected with both Ab-175 and mAb7a antibodies. No submembranous localization of gephyrin micro-clusters was observed. Scale bar, 15 μm

To provide further evidence for the inhibition of gephyrin and MT binding by CbII-mediated gephyrin phosphorylation, we analyzed the subcellular distribution of gephyrin in the absence or presence of CbII in various cell types using fluorescence microscopy. Upon expression in HEK293T cells, gephyrin detected with the anti-gephyrin antibody Ab-175 disclosed a cytoplasmic localization (Fig. 4a), consistent with a putative binding of gephyrin to MTs. This finding was supported also by the electron microscopy of immunogold labelled LRW-embedded HEK293T cells showing gephyrin aggregates near the nucleus and mitochondria close to MT structures, thus sustaining a colocalization of MT with gephyrin (suppl. Figure 2).

In IF images the distribution of Ab-175-positive gephyrin immunoreactivity was either diffusely or in small clusters in the cytoplasm of HEK293T cells and was dependent on the duration of gephyrin expression. From 18 to 24 h post-transfection we observed an increase in the number of small clusters, whereas two days after transfection large intracellular aggregates (bodies) were formed (Kirsch et al. 1995; Harvey et al. 2004) (48 h) (Fig. 4a). In a double-labeling experiment using Ab-175 and mAb7a antibodies, mAb7a immunoreactivity was barely found in the cytoplasm and didn't overlap with dispersed cytoplasmic gephyrin labeled with Ab-175 (Fig. 4a) in HEK293T cells 18 or 24 h after transfection. However, some but not all large gephyrin bodies in cells at 48 h post-transfection displayed a weak mAb7a immunofluorescence signal (Fig. 4a). Moreover, we detected mAb7a immunoreactivity in nuclei, an observation which may need further investigation.

To avoid the formation of non-physiological gephyrin aggregates due to protein overexpression observed upon 48 h expression, for the following experiments cells 24 h post transfection were used.

We co-transfected HEK293T cells with gephyrin and CbI or CbII lacking a N-terminal SH3 domain (CbII_(-SH3)_) and performed triple immunolabeling with Ab-175, mAb7a and anti-Cb antibodies. As expected, the detection of gephyrin with mAb7a was profoundly altered in the presence of CbII lacking the SH3 domain, increased mAb7a immunoreactivity being detected mostly in small clusters next to the plasma membrane (Fig. 4b). In contrast, in HEK293T cells expressing recombinant gephyrin and CbI, only cytoplasmic "bodies", that overlapped with Ab-175 and mAb7a immunolabelling, were observed, whereas the dispersed cytoplasmic gephyrin immunoreactivity seen with Ab-175 was not labeled using mAb7a. Thus, gephyrin that is assembled in either sub-membranous smaller micro-clusters or small and larger cytoplasmic aggregates binds mAb7a antibodies, whereas dispersed cytoplasmic, possibly MT bound gephyrin, is not detected with mAb7a, thus supporting our hypothesis, that gephyrin bound to MTs is not phosphorylated at sites detected with mAb7a.

### Gephyrin and MTs are co-localized in U2OS cells

**Fig. 5:**
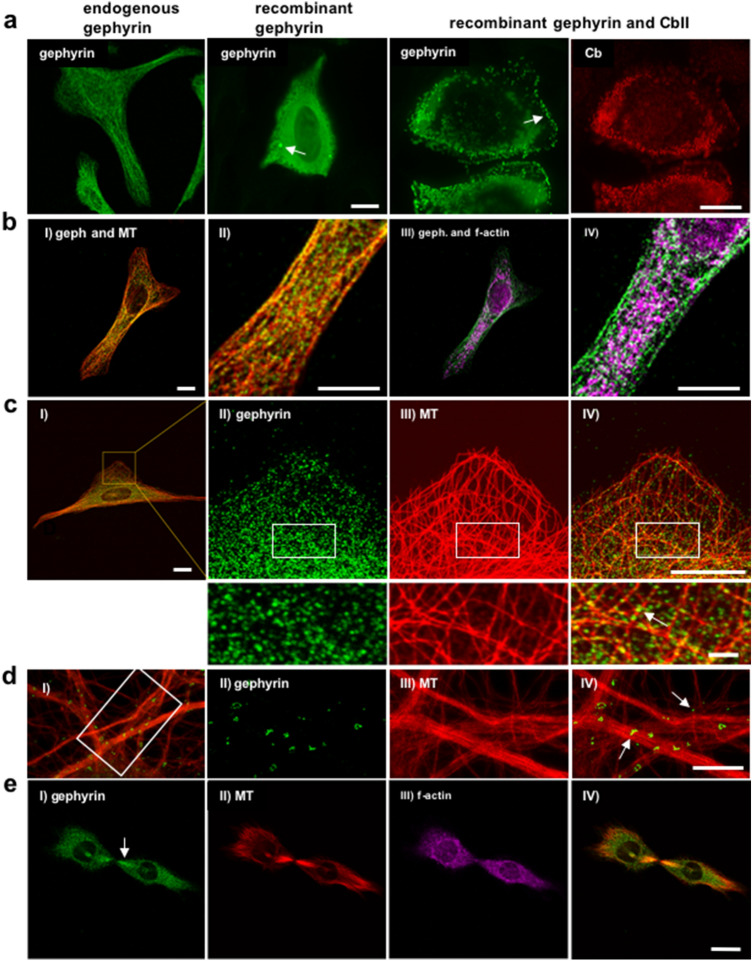
Co-localisation of gephyrin with MTs in U2OS cells. **a** Subcellular distribution of gephyrin in U2OS cells. Immunofluorescence microscopy of U2OS cells stained with anti-gephyrin antibody Ab-175 demonstrated punctuated and filamentous endogenous gephyrin distribution in non-transfected cells and diffusely distributed recombinant gephyrin with small aggregates (arrow) 24 h post-transfection, scale bar is 15 μm. Similar to HEK293T cells, both Ab-175 and Cb immunoreactivity redistributed from the cytoplasm to submembrane micro-clusters (arrow) upon co-transfection (right panel). Scale bar is 15 μm. **b** Gephyrin co-localized with MTs rather than with F-actin. Subcellular distribution of endogenous gephyrin, MTs and F-actin was visualized using Ab-175, DM1α and phalloidin, respectively. I) Merge of gephyrin and MT staining, scale bar is 15 μm. II) Enlargement of I, scale bar is 15 μm. III) merge of gephyrin and F-actin labelling, IV) enlargement of III. **c** Images of another U2OS cell. This cell displays larger lamellipodia-like structures revealing the co-localization of Ab-175 positive gephyrin immunoreactivity with MTs. Subcellular distribution of endogenous gephyrin and MTs were visualized using Ab-175 and anti-tubulin antibody DM1α, respectively. Punctuated and clustered Ab-175 immunoreactivity overlapped with DM1α staining. I) Merge of gephyrin and MT staining, scale bar is 15 μm. II) Enlargement of lamellipodia with gephyrin staining, III) MT-staining, IV) merge of II and III, scale bar is 15 μm. Rectangles define regions of corresponding enlargements shown below with co-localized gephyrin and MTs indicated by an arrow. Scale bar is 2 μm. **d** STED- images of another U2OS cell with punctuated Ab-175 immunoreactivity overlapping with MTs. I) Overlay with a rectangle delimiting enlargements shown at the right side. II) Gephyrin puncta, III) MT staining. III) Overlay revealing small gephyrin puncta colocalized with MT (arrows). **e** Colocalization of gephyrin and MTs in midbody structures. Immunofluorescent microscopy using Ab-175 and DM1α showed strong co-localization of gephyrin with both fiber-like bundled microtubules and midbody (arrow) during cell mitosis. I) Gephyrin stained with Ab-175, II) MT, III) F-actin, IV) merged images. Scale bar is 15 μm

To further analyze the putative association of gephyrin and MT we employed the cell line U2OS for additional experiments with fluorescence microscopy. Upon staining U2OS cells with Ab-175, we observed a more dispersed filament-like and micro-clustered structure of endogenous gephyrin compared to cells expressing recombinant gephyrin (Fig. 5a). In accordance with our finding in HEK293T cells, gephyrin immunoreactivity detected with Ab-175 was re-localized from the cytoplasm to small sub-membranous clusters when co-expressing gephyrin and CbII_(SH3-)_ (Fig. 5a, right panel). To avoid overexpression artifacts, we decided to analyze the subcellular localization of endogenous gephyrin and MTs in U2OS. Interestingly, triple staining in non-transfected U2OS cells revealed a filamentous and punctuated appearance of the Ab-175 immunoreactivity (gephyrin) strongly overlapping with MTs appearing as filaments labeled by DM1α, but only to a minor degree with phalloidin labeled actin filaments (Fig. 5b, c). These gephyrin structures were not detected with mAb7a (data not shown). Specifically, using a STEDYCON microscope allowing a resolution to about 30 nm, we observed tiny particle-like gephyrin immunoreactivities with a size comparable to a MT diameter, and arranged along filamentous MTs (Fig. 5d). In addition, we observed the co-localization of gephyrin- and tubulin- immunoreactivity in the midbody of dividing U2OS-cells (Fig. 5e).

## Discussion

We have earlier shown that gephyrin, the main organizer of ligand-gated ion channels at inhibitory synapses interacts at post-synaptic densities with the MT-based cytoskeleton (Kirsch et al. 1991; Kirsch and Betz 1995). In this study, we provide evidence that the binding of gephyrin to MT is dependent on posttranslational modifications of gephyrin i.e. the phosphorylation state of this multifunctional protein. Firstly, we demonstrated that a fraction of gephyrin was copurified with tubulin from rat brain extracts and disclosed that gephyrin highly phosphorylated at Ser270 was not bound to polymerized MTs. Secondly, fostering the phosphorylation of gephyrin at Ser270 by co-expressing CbII_-SH2_ in HEK293T cells the binding of gephyrin to MTs was reduced. Thirdly, expressing gephyrin carrying alanine substitutions at Ser270, Ser198 and Ser200 abolished this effect. Finally, the analysis of the subcellular localization of endogenous gephyrin in HEK293T cells and U2OS cells clearly revealed a co-localization of gephyrin with MTs, though this gephyrin was not phosphorylated at Ser270.

Altogether these findings suggest that phosphorylation of gephyrin negatively regulate its binding to MTs, pointing to an already well-known phenomenon for other MAPs like tau which detaches from MTs upon defined phosphorylation states (Ramkumar et al. 2018).

Purification of tubulin from brain tissue through repeated cycles of polymerization and depolymerization is a long-established method used for in vitro studies of MAPs (Sloboda 2015). To support our observation of gephyrin co-purification with polymerized tubulin from rat brains, we performed MT co-sedimentation assays also with recombinant gephyrin expressed in HEK293T cells under different conditions known to affect tubulin polymerization. Importantly, upon elevated MT polymerization at higher tubulin or GTP and paclitaxel concentrations, we detected increasing amounts of gephyrin in the MT-rich pellet supporting our initial finding that gephyrin binds with a predilection to polymerized MT rather than soluble tubulin in vitro (Kirsch et al. 1991).

Different studies have shown the important roles of gephyrin-MT interactions in postsynaptic cluster formation (Groeneweg et al. 2018). Particularly, MTs were required for synaptic localization of GlyRs in young cultured spinal neurons (Kirsch and Betz 1995; van Zundert et al. 2004). However, the molecular basis of gephyrin-cytoskeleton interactions and how these interactions affect the gephyrin-mediated receptor anchoring is still poorly understood. Recently, evidence has been provided for posttranslational modifications of gephyrin, especially phosphorylation, modulating the size and density of postsynaptic gephyrin and thus clustering of inhibitory ligand-gated ion channels (Flores et al. 2015; Ghosh et al. 2016). But whether the phosphorylation regulates the organization of postsynaptic receptors through modifying gephyrin-MT interactions remains unknown.

We employed the anti-gephyrin antibody mAb7a to detect phospho-gephyrin in brain tubulin preparations. Our previous study showed that mAb7a is a phospho-specific antibody that allows the cellular and biochemical analysis of gephyrin phosphorylation at Ser270 (Kuhse et al. 2012). Moreover, the gephyrin binding protein Cb is involved in gephyrin phosphorylation which can be recognized by mAb7a (Kuhse et al. 2012). Surprisingly, in all rat brain extracts tested, we detected mAb7a- specific immunoreactivity exclusively in the soluble protein fraction. Consistently, upon co-transfection of HEK293T cells with CbII_-SH2_, we found a robust increase of phosphorylated gephyrin signal in the supernatant using MT co-sedimentation assay. Furthermore, co-expressing a gephyrin mutant lacking the phosphorylation sites Ser198, Ser200 and Ser270 with CbII_-SH2_ gephyrin binding to MTs compared to wild type was increased. These corresponding and complementary results of brain tissue and HEK293T cell extracts experiments support our hypothesis that phosphorylated gephyrin is unlikely to bind to the MT and pinpoint this posttranslational modification as an effective molecular mechanism regulating gephyrin—MT association.

Mass spectrometry analysis has identified 22 phosphorylation sites on gephyrin. Most of them are located within the C-domain (Herweg and Schwarz 2012) positioned between the highly conserved G- and E-domains that are directly involved in gephyrin multimerization and contribute to the recruitment of Cb (Papadopoulos et al. 2007). Conformational changes in this region induced by phosphorylation could affect the folding of the C-domain itself and of the neighboring G- and E-domains, thus altering gephyrin properties (Herweg and Schwarz 2012). The antibody mAb7a is directed against an epitope including Ser270 in the C-domain, shown to be targeted by both the proline directed CDK5 (Kalbouneh et al. 2014) and GSK-3β (Tyagarajan et al. 2011). Phosphorylation of gephyrin at Ser270 by GSK-3β was reported to exert a negative effect on gephyrin clustering at GABAergic synapses (Tyagarajan et al. 2011). Knockdown or inhibition of CDK5 (Kalbouneh et al. 2014) or the CDK5 activator p35 (Kiss et al. 2020) in hippocampal neurons resulted in reduced phosphorylation of postsynaptic gephyrin immunoreactivity and a decrease in the number of γ2 containing GABA_A_R clusters.

We also investigated the impact of co-expressed Cb-isoforms on the subcellular localization and phosphorylation of recombinant gephyrin in HEK293T cells. These experiments confirmed earlier studies (Kins et al. 2000) demonstrating the re-localization of gephyrin to the cell membrane upon co-expressing CbII_-SH2_ but not CbI. These observations sustain the hypothesis that phosphorylated gephyrin removed from a MT bound stage in the cytoplasm could be moved to the plasma membrane and might be attached to it by Cb which binds to phospho-inositol-3-phosphate with its PH domain (Kins et al. 2000; Soykan et al. 2014).

Taking advantage of the extended cytosolic area of U2OS cells, using triple -immunolabelling and confocal/STED- microscopy we could clearly observe that the mostly punctuated structures of gephyrin largely overlapped with MTs, but only sparsely with F-actin. These small punctuated structures were smaller than the gephyrin clusters at postsynaptic membrane specializations and could well correspond to the predominant hexameric form of gephyrin composed from trimers (Sander et al. 2013), which we have detected in U2OS protein extracts using blue-native gel-electrophoresis.

Similar pattern of MTs bound gephyrin complexes in neurons was observed also by others (Maas et al. 2006). Using GFP-gephyrin fusion protein to visualize gephyrin-containing complexes by fluorescence microscopy Maas et al. demonstrated that GlyR–gephyrin–dynein complexes are retrogradely transported along MTs in cultured neurons (Maas et al. 2006, 2009; Hanus et al. 2004).

Our experimental methods do not allow to distinguish between direct or indirect binding of gephyrin to microtubules. However, our results indicate that gephyrin phosphorylation detectable by mAb7a staining might regulate the association and dissociation of gephyrin with MT-dependent co-transport complexes. Further studies are necessary to investigate whether the MT-based transport of receptors and gephyrin and their subsequent anchoring at postsynaptic sites are indeed dependent on the phosphorylation state of gephyrin.

Interestingly, we also observed that endogenous gephyrin colocalized with tubulin in the midbody of dividing U2OS cells. This observation, together with the findings of another study revealing intracellular gephyrin aggregates highly colocalized with MT-organizing center (MTOC) structures (Fuhrmann et al. 2002), point to the gephyrin-MT association as a target of further investigations also in the context of cell division.

## Supplementary Information

Below is the link to the electronic supplementary material.

Supplementary file 1

## Data Availability

The authors declare the availability of all data and materials of the manuscript.
