# Supplementary material for: Binding of gephyrin to microtubules is regulated by its phosphorylation at Ser270
Source: Histochem Cell Biol. 2021 Apr 1;156(1):5–18. doi: 10.1007/s00418-021-01973-2 (PMC8277605; doi:10.1007/s00418-021-01973-2)
Supplement: Supplementary file 1 [file 418_2021_1973_MOESM1_ESM.docx]

**Suppl. Material**

**Suppl. Figure 1:**

Gephyrin expressed in HEK293T or U2OS cells forms soluble high molecular weight oligomers very similar to liver or brain tissue.


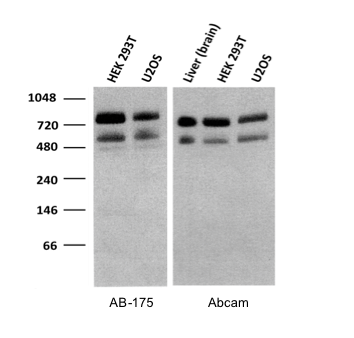


Legend Suppl. Figure 1. Gephyrin expressed in HEK293T or U2OS cells forms soluble high molecular weight oligomers very similar to brain tissue.

Proteins were extracted from HEK293T-, U2OS cells and mouse liver tissue. Immunoblot analysis using blue-native polyacrylamide gel electrophoresis was performed. Anti-gephyrin antibody Ab-175 detected two bands around the size of 800kD and 600kD in HEK293T and U2OS cells. Using another pan-anti-gephyrin antibody (Abcam), a similar pattern was detected in HEK293T, U2OS cells and liver tissue.

**Suppl. Figure 2:**

Ultrastructure of recombinant gephyrin aggregates in a HEK293T cell.


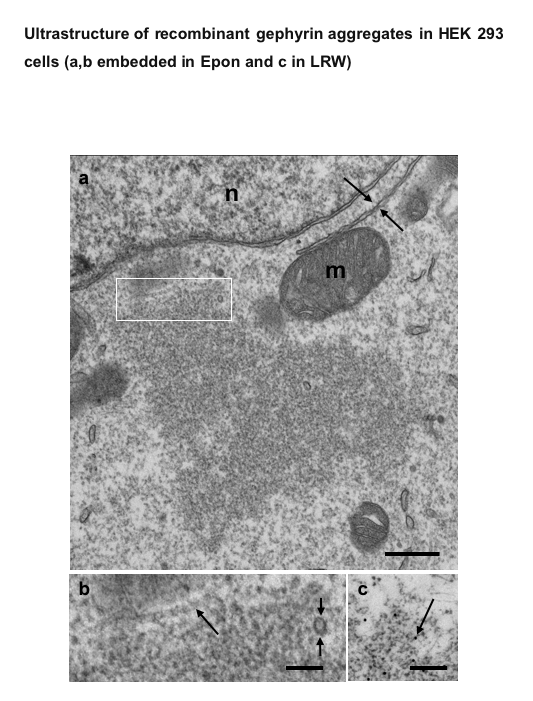


Legend Suppl. Figure 2

a) Transmission electron microscope image showing the inside of a HEK293T cell. The protein aggregates (gephyrin) were detected mostly adjacent to the cell nucleus (n) and often to mitochondria (m). Two arrows show to a cisterna of the ER. The rectangle indicates the region (enlarged in b), where a microtubulus is seen in direct neighborhood to the gephyrin aggregate. b) The enlargement of the longitudinally sectioned microtubule (arrow) reveals its decoration with proteins. Two arrows show to a vesicle of ER c) Immunogold labeling of gephyrin within a peripheral aggregate region. The arrow marks gephyrin labeled with a 15 nm Au-particle. The bars represent 500nm in a) and 100nm in b) and c).
